# Are Vegetarian and Vegan Diets Associated With Eating Disorder Symptoms? A Systematic Review and Meta‐Analysis

**DOI:** 10.1002/eat.70158

**Published:** 2026-07-07

**Authors:** Valentina Díaz‐Goñi, Arthur Eumann Mesas, Estela Jiménez‐López, Tomás Olivo‐Martins‐de‐Passos, Fernando Peral‐Martínez, Ana María Peraile‐Huerta, María Eugenia Visier‐Alfonso, Miriam Garrido‐Miguel, Bruno Bizzozero‐Peroni

**Affiliations:** ^1^ Health and Social Research Center Universidad de Castilla‐La Mancha Cuenca Spain; ^2^ Institute for Health Research of Castilla‐La Mancha (IDISCAM) Toledo Spain; ^3^ Centro de Investigación Biomédica en Red de Salud Mental (CIBERSAM) Instituto de Salud Carlos III Madrid Spain; ^4^ Faculty of Nursing Universidad de Castilla‐La Mancha Albacete Spain; ^5^ Aging Research Center, Department of Neurobiology, Care Sciences and Society Karolinska Institutet and Stockholm University Stockholm Sweden; ^6^ Higher Institute of Physical Education Universidad de la República Maldonado Uruguay

**Keywords:** dietary patterns, disordered eating behavior, feeding and eating disorders, mental disorders, pathological eating, plant‐based diet

## Abstract

**Objective:**

To synthesize the evidence on the associations between vegetarian and vegan diets (VVDs) and eating disorder (ED) symptoms compared with omnivorous diets across the lifespan.

**Method:**

Following the preferred reporting items for systematic reviews and meta‐analyses (PRISMA) 2020 Guidelines, we searched PubMed, Scopus, Cochrane Library, PsycINFO, and Web of Science Databases up to May 1, 2026, with no language or date restrictions. Random effects models with the Hartung–Knapp–Sidik–Jonkman method were used to estimate pooled effect sizes (standardized mean difference [SMD] with 95% confidence interval [CIs]).

**Results:**

We included 24 cross‐sectional studies with a total of 25,223 participants (67.7% female; mean age range: 14.9–49.6 years). ED symptoms did not differ significantly between participants who followed VVDs and those following omnivorous diets in analyses including all eligible studies (SMD = 0.07; 95% CI: −0.04 to 0.19; *I* ^2^ = 80.3%; 95% prediction interval: −0.44 to 0.59). Results were unchanged when analyses were restricted to adult populations. No significant differences in ED symptoms were observed according to diet type (*p* = 0.524), measurement scale (*p* = 0.392), or geographic region (*p* = 0.633). In analyses restricted to adults, sex significantly moderated the pooled estimates (*p* = 0.028), with studies including a higher proportion of females reporting lower ED symptom scores among individuals following VVDs compared with omnivores.

**Discussion:**

ED symptom levels do not appear to differ significantly between individuals following VVDs and those following omnivorous diets. Longitudinal studies accounting for dietary quality, duration of adherence, dietary motivations, and psychosocial factors are also needed to clarify this association.

## Summary

- This is the first meta‐analysis to quantify the association between vegetarian and vegan diets and eating disorder (ED) symptoms compared with omnivorous diets across the lifespan.
- No statistically significant differences in ED symptoms were observed between dietary groups. The available evidence was predominantly derived from young and middle‐aged adults.
- Sex significantly moderated the pooled estimates in analyses restricted to adult populations, with studies including a higher proportion of females showing lower ED symptom scores among those following vegetarian or vegan diets.
- Findings should be interpreted cautiously due to substantial heterogeneity and the cross‐sectional nature of the evidence. No longitudinal evidence was identified, highlighting the need for studies examining directionality of associations, particularly in adolescent and older populations.

## 1 Introduction

Eating disorders (EDs) are serious psychiatric conditions characterized by persistent disturbances in eating behaviors and weight regulation, often resulting in severe physical and psychological consequences (American Psychiatric Association 2022). They represent a growing global public health concern worldwide, affecting millions of individuals and contributing to the global burden of disease (GBD 2023 Mental Disorder Collaborators. 2026; Treasure et al. 2020). Although EDs most often emerge during adolescence, they may persist, recur, or first manifest later in life, resulting in important clinical, functional, and public health consequences across the lifespan (Brandsma 2007; Khalsa et al. 2017; Södersten et al. 2006).

The etiology of EDs is multifactorial and dynamic, involving the interaction of genetic vulnerability, neurobiological mechanisms (Kaye et al. 1998, 2005), psychological traits, and environmental influences (Becker et al. 2011; Erriu et al. 2020; Hesse‐Biber et al. 2006). Moreover, underdiagnosis and undertreatment remain common due to the hidden nature of symptoms and the stigma surrounding help‐seeking (Treasure et al. 2020). A thorough understanding of factors associated with ED symptoms is, therefore, essential to inform effective public health, clinical, and educational strategies aimed at preventing and reducing the burden of EDs (Horovitz and Argyrides 2023).

In this context, attention has increasingly focused on socially accepted dietary patterns characterized by food restriction or exclusion, such as vegetarian and vegan diets (VVDs) (Gilbody et al. 1999). VVDs are plant‐based diets that vary from the exclusion of any type of meat (i.e., ovo‐vegetarian, lacto‐vegetarian, and ovo‐lacto‐vegetarian diets) to the exclusion of all animal‐derived foods or food products (i.e., vegan diets) (Hargreaves et al. 2023). The prevalence of VVDs has increased worldwide in recent decades, particularly among women and younger adults (Nezlek and Forestell 2024). Although individuals may adopt these diets for a variety of reasons, such as religious, ethical, health, or environmental concerns (Kim et al. 2022; Mazzolani et al. 2023; Rosenfeld 2018), growing attention has been directed toward their possible association with EDs. Some authors suggested that VVD patterns may provide a socially acceptable framework for restrictive eating behaviors associated with ED psychopathology, particularly when adopted for weight‐control purposes (Bardone‐Cone et al. 2012). Conversely, individuals with EDs may be more likely to adopt vegetarian dietary patterns, raising the possibility of reverse causality in the observed associations (Heiss, Hormes, and Timko 2017; Mathieu et al. 2023). Nevertheless, current evidence is inconsistent, and it remains unclear whether adherence to a VVD is associated with increased ED symptomatology (Heiss, Hormes, and Timko 2017; Mathieu et al. 2023).

Previous systematic reviews have reported mixed and inconclusive findings. Specifically, while in a review higher ED symptom levels were associated with higher VVD adherence among adolescents and young adults (Sergentanis et al. 2020), two other reviews reported no clear associations after examining studies in adults (Mathieu et al. 2023; McLean et al. 2022a). Furthermore, these reviews often classified dietary patterns as vegetarian even when they included occasional meat or fish consumption (e.g., pescatarian, semi‐vegetarian, or flexitarian diets), which may have limited the interpretability of the findings. Since these dietary patterns differ substantially in food consumption and motivations for adherence, combining them in a single exposure category may obscure associations specific to strict VVDs. Importantly, no previous meta‐analysis has comprehensively evaluated the association between adherence to strict VVDs (i.e., dietary patterns that completely exclude all types of meat, including poultry and seafood) (Hargreaves et al. 2023) and ED symptoms in community‐based populations. To address this gap, the present systematic review and meta‐analysis aimed to synthesize the available evidence and estimate this association across the lifespan.

## 2 Method

This systematic review with meta‐analysis was conducted according to the Cochrane Handbook for Systematic Reviews (Higgins et al. 2024) and the 2020 Preferred Reporting Items for Systematic Reviews and Meta‐Analyses (PRISMA) (Page et al. 2021). The protocol of the systematic review was registered in PROSPERO (reference number: CRD42024527351). During the review process, a protocol amendment was made to remove the age restriction originally specified in the PROSPERO registration (participant age ≥ 18 years), thereby allowing the inclusion of studies across all age groups. Two researchers (VD‐G and BB‐P) independently performed the literature search, screening, study selection, data extraction, and methodological quality assessment. Discrepancies were resolved by consensus with a third researcher (AEM).

### 2.1 Search Strategy

A systematic search was conducted in the following electronic databases, from their inception to October 9, 2025: PubMed, Scopus, Cochrane Library, PsycINFO, and Web of Science. The search was subsequently updated to include the period from October 10, 2025, to May 1, 2026. Gray literature searches were also conducted using Google Scholar to identify potentially relevant peer‐reviewed studies not captured in the primary databases. Additionally, backward citation searches were performed by reviewing the reference lists of relevant systematic reviews (Mathieu et al. 2023; McLean et al. 2022a; Sergentanis et al. 2020) and all included studies. The search strategy included terms related to the study objective, such as “vegetarian”, “vegan”, “plant‐based”, and “eating disorder”. The complete search strategy for all databases is provided in Table S1.

### 2.2 Eligibility Criteria

To be included, studies had to meet the following criteria: (i) population: nonclinical populations (i.e., participants not recruited or selected on the basis of a diagnosed physical or mental health condition); (ii) exposure: strict vegetarian (i.e., lacto‐vegetarian, ovo‐vegetarian, lacto‐ovo‐vegetarian) and/or vegan (i.e., exclusively plant‐derived foods) diets, according to the Academy of Nutrition and Dietetics (Melina et al. 2016) and other commonly used definitions in the literature (Hargreaves et al. 2023); (iii) comparison: omnivorous diets that encompassed the consumption of any type of meat; (iv) outcome: ED psychopathology assessed using validated observer‐rated or self‐report instruments providing a global measure of ED symptoms. Eligible outcomes also included categorical outcomes based on established cut‐off points and ED diagnoses identified through self‐report or clinical assessment within community‐based populations; and (v) study design: observational studies (i.e., cross‐sectional and longitudinal) published in peer‐reviewed academic journals.

Studies were excluded if they focused on clinical or special populations (e.g., anorexia, depression, athletes, pregnant individuals), misclassified VVDs (i.e., not explicitly defined or included meat eaters such as pescatarians or flexitarians), or compared VVDs with other special diets (e.g., gluten free, paleo). No language, publication date or other restrictions were applied.

### 2.3 Study Selection

All identified studies were uploaded to the Rayyan online review platform (Ouzzani et al. 2016) and deduplicated. Titles and abstracts were screened, and potentially eligible studies were subsequently retrieved to full‐text review to verify compliance with the predefined eligibility criteria.

### 2.4 Data Extraction

The following data were extracted from each study: (i) first author surname, publication year and country; (ii) sample characteristics, including total sample size, mean age, sex assigned at birth and/or gender, race and/or ethnicity, socioeconomic status, body mass index (BMI), educational level (percentage of any university degree) and subsamples according to diet type; (iii) dietary exposure, including vegetarian, vegan, or mixed (vegetarian plus vegan); (iv) measure of ED symptoms, including validated observer‐rated or self‐report instruments providing a global score of ED psychopathology, categorical outcomes based on established cut‐off points from these instruments, and ED diagnoses identified through self‐report or clinical assessment; and (v) outcome data, including global scores and measures of variability (e.g., standard deviations [SD], standard errors), frequencies, effect estimates, or other information required to calculate effect size.

### 2.5 Methodological Quality Assessment

The methodological quality of the included studies was assessed using the National Institutes of Health (NIH) Quality Assessment Tool for Observational Cohorts and Cross‐Sectional Studies (National Heart Lung and Blood Institute 2021). This instrument includes 14 items covering domains such as study population, timeframe for study associations, exposure and outcome measures, and statistical analysis adjustments. Each item was rated as yes, no, not reported, not applicable, and cannot be determined. Based on these ratings, overall quality was judged according to the NIH quality rating guide (National Heart Lung and Blood Institute 2021), classifying each cross‐sectional study as good, fair, or poor.

### 2.6 Effect Size

Standardized mean difference (SMD) using Cohen's *d* index (Cohen 1988) was applied as the main effect size. The means, SDs, and sample sizes for each diet group were extracted from the included studies to estimate SMDs. Global scores from validated ED instruments were extracted to maximize comparability across studies. When outcome data were reported as standard errors (Zickgraf et al. 2020), frequencies (Gwioździk et al. 2022; Köse et al. 2021; Perry et al. 2001), beta coefficients (Collins and Quinton 2020), or medians with interquartile ranges (Białek‐Dratwa et al. 2024), the appropriate conversion formulas were applied to estimate SMDs (Higgins et al. 2024; McGrath et al. 2020). Negative SMD values indicate that individuals adhering to VVDs reported lower overall levels of ED symptoms than those following omnivorous diets. The criteria proposed by Cohen were used to interpret the effect size as small (SMD = 0.2), moderate (SMD = 0.5), or large (SMD = 0.8) (Cohen 1988).

### 2.7 Data Synthesis

A meta‐analysis of cross‐sectional studies comparing ED symptoms of VVDs with those of omnivorous diets was conducted via a random effects model with the Hartung–Knapp–Sidik–Jonkman method (Inthout et al. 2014). The analyses were performed when more than two studies analyzed the outcome (Jackson and Turner 2017). Heterogeneity across studies was assessed using the *I* ^2^ statistic, with values of 0%–40% indicating not important, 30%–60% moderate, 50%–90% substantial, and 75%–100% considerable (Higgins et al. 2024). The corresponding *p* values were also considered. In addition, 95% prediction intervals were estimated to reflect the expected range of SMDs in future studies. Primary analyses included all eligible studies, encompassing both adolescent and adult populations. Given that most included studies were conducted in adults, all analyses were repeated in studies restricted to adult populations (i.e., excluding studies with participants younger than 18 years).

Subgroup analyses were conducted according to diet type (vegetarian or vegan diets), the self‐reported scales applied and geographic region. Univariate meta‐regression models were conducted to examine whether participant and study characteristics (age [years], sex [% female], total sample size, BMI [kg/m^2^], underweight status [% with BMI < 18 kg/m^2^], educational level [% with any university degree], and publication year) influenced the pooled SMD and between‐study heterogeneity. Sensitivity analyses were performed to assess the robustness of the summary estimates using the leave‐one‐out method (Higgins et al. 2024). Publication bias was evaluated through Egger's regression test for funnel plot asymmetry and visual inspection of funnel plots (Sterne et al. 2001). Statistical significance was set at *p* < 0.05. All analyses were conducted using R (version 4.2.3; R Foundation for Statistical Computing, Vienna, Austria) with the meta (Balduzzi et al. 2019) package.

### 2.8 Additional Methodological Considerations

If a study reported data from different scales (McLean et al. 2021, 2022, 2023) or from different motivations for following a vegetarian diet (Zickgraf et al. 2020), the corresponding SMDs were pooled into a single estimate for the main analysis. When studies provided separate data for vegetarians and vegans (Barnett et al. 2016; Coimbra and Ferreira 2021; Collins and Quinton 2020; Hanras et al. 2022; McLean et al. 2022, 2023; Norwood et al. 2019; Timko et al. 2012), these results were included in their respective subgroup analyses. In such cases, data on ED symptoms were also combined to calculate a single pooled SMD for inclusion of each study in the main meta‐analysis (VVDs vs. omnivorous diets). For one study that did not report SD, the SD was assumed to be equal to the reported mean to enable calculation of the effect size (Norwood et al. 2019).

## 3 Results

### 3.1 Study Selection

A total of 780 records were identified, of which 47 were assessed in full text for eligibility and 24 were ultimately excluded for various reasons (Table S2). In the search update, 105 additional records were identified; after removal of duplicates, 62 records were screened by title and abstract, 7 articles were assessed in full text, and 1 additional study met the inclusion criteria. Finally, a total of 24 cross‐sectional studies (Barnett et al. 2016; Białek‐Dratwa et al. 2024; Çiçekoğlu and Tunçay 2018; Coimbra and Ferreira 2021; Collins and Quinton 2020; Dorard and Mathieu 2021; Forestell et al. 2012; Gorgoni et al. 2026; Gwioździk et al. 2022; Hanras et al. 2022; Heiss, Coffino, and Hormes 2017; Heiss et al. 2019; Köse et al. 2021; Lindeman 2002; Lindeman et al. 2000; McLean et al. 2021, 2022, 2023; Norwood et al. 2019; Paslakis et al. 2020; Sieke et al. 2021; Timko et al. 2012; Zickgraf et al. 2020) were included in the systematic review and meta‐analysis (Figure 1).

**FIGURE 1:**
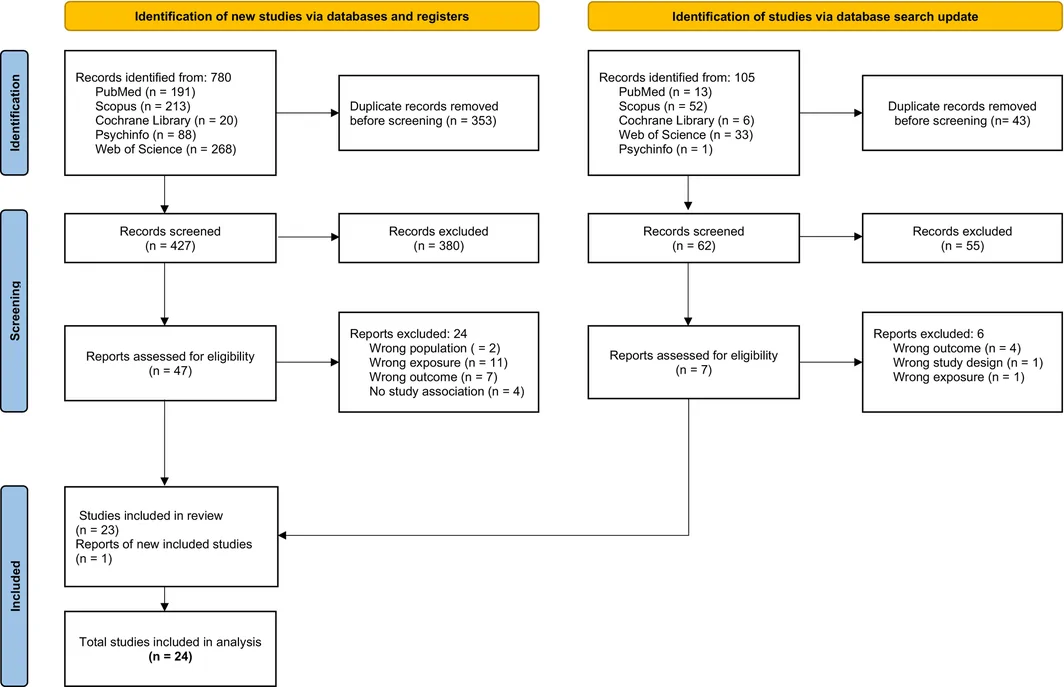
PRISMA flow diagram of study selection.

### 3.2 Characteristics of Studies and Participants

Table 1 shows the main characteristics of the studies included, published between 2000 (Lindeman et al. 2000) and 2026 (Gorgoni et al. 2026). The total sample included 25,223 individuals (67.7% females), with mean ages ranging from 14.9 (Perry et al. 2001) to 49.6 (Paslakis et al. 2020) years and mean BMIs from 22.0 (Köse et al. 2021) to 25.9 (Paslakis et al. 2020) kg/m^2^. Four studies included both adolescent (< 18 years) and adult (≥ 18 years) participants (Collins and Quinton 2020; Lindeman 2002; Lindeman et al. 2000; Perry et al. 2001), whereas the remaining studies were conducted exclusively in adults. Underweight status varies from 3.6% (Collins and Quinton 2020) to 15.6% (Białek‐Dratwa et al. 2024). The participants were recruited from different countries, including Australia (Collins and Quinton 2020; McLean et al. 2021, 2022, 2023; Norwood et al. 2019), Belgium (Dorard and Mathieu 2021), Canada (Dorard and Mathieu 2021; McLean et al. 2021), Finland (Lindeman 2002; Lindeman et al. 2000), France (Dorard and Mathieu 2021; Hanras et al. 2022), Germany (Paslakis et al. 2020), Italy (Gorgoni et al. 2026), Poland (Białek‐Dratwa et al. 2024; Gwioździk et al. 2022), Portugal (Coimbra and Ferreira 2021), Turkey (Çiçekoğlu and Tunçay 2018; Köse et al. 2021), United Kingdom (UK) (McLean et al. 2021) and United States of America (USA) (Barnett et al. 2016; Forestell et al. 2012; Heiss, Coffino, and Hormes 2017; Heiss et al. 2019; McLean et al. 2021; Perry et al. 2001; Sieke et al. 2021; Timko et al. 2012; Zickgraf et al. 2020).

**TABLE 1:** Characteristics of the included studies.

Author, year, country	*N* (Total)	Age (mean years)	Sex assigned at birth and/or gender (%)	Race/ethnicity (*n*)	SES	BMI (mean)	Education level (% with any university education)	Dietary exposure (*n*)	Measure of ED symptoms
Barnett et al. (2016), USA	284	38.0	Female = 83.4 male = 16.6	NR	NR	24.9	26.6	Vegetarians: 22 vegans: 11 omnivores:213	EDE‐Q
Białek‐Dratwa et al. (2024), Poland	186	NR	Female = 94.0 male = 5.9	NR	NR	NR	51.1	Vegetarians/vegans: 84 omnivores: 102	EAT‐26
Çiçekoğlu and Tunçay (2018), Turkey	62	34.0	Female = 61.0	NR	NR	NR	96.7	Vegetarians/vegans: 31 omnivores: 31	EAT‐40
Coimbra and Ferreira (2021), Portugal	447	32.7	Female = 100	NR	NR	23.8	NR	Vegetarians: 61 vegans: 50 omnivores: 281	EDE‐Q
Collins and Quinton (2020), Australia	634	28.8	Female = 100	Caucasian non‐Hispanic (578) Hispanic (6) Asian (18) Aboriginal/Torres Strait Islander (11) Middle Eastern (6) Pacific Islander (2) Black or African American (2) other (11)	NR	24.9	NR	Vegetarians: 103 vegans: 280 omnivores: 212	EAT‐26
Dorard and Mathieu (2021), France, Belgium, and Canada	101	29.5	Female = 74.3 male = 25.7	NR	NR	22.9	50.0	Vegetarians/vegans: 49 omnivores: 52	EAT‐26
Forestell et al. (2012), USA	240	19.2	Female = 100	Caucasian (115) Asian (18) African American (9) mixed (more than one race) (4)	Family income, > $75,000. *n* = 95	22.0	100	Vegetarians/vegans: 55 omnivores: 91	EAT‐26
Gorgoni et al. (2026), Italy	747	36.4	Female = 73.8 male = 26.2	NR	NR	23.2	NR	Vegetarian/vegan: 215 omnivores: 532	EDE‐Q
Gwioździk et al. (2022), Poland	420	24.0	Female = 100	NR	NR	NR	88.1	Vegetarians: 115 omnivores: 227	SCOFF
Hanras et al. (2022), France	589	31.8	Female = 83.4	NR	Student (27.7%) employed (56.4%) unemployed (14.1%) retirement (1.9%)	24.7	74.0	Vegetarians: 112 vegans: 127 omnivores: 212	EAT‐26
Heiss, Coffino, and Hormes (2017), USA^a^	577	30.6	Female = 80.4	White (498) American Indian/Alaska Native (11) Asian/Pacific Islander (33) Black or African American (12) Other (5)	NR	24.3	NR	Vegans: 357 omnivores: 220	EDE‐Q
Heiss et al. (2019), USA^a^	381	31.1	Female = 80.8	White (308)	NR	25.2	NR	Vegetarians: 50 omnivores: 106	EDE‐Q
Köse et al. (2021), Turkey	318	21.6	Female = 77.4 male = 22.6	NR	NR	22.0	100	Vegetarians/vegans: 47 omnivores: 242	EAT‐40
Lindeman et al. (2000), Finland^b^	118	16.3	Female = 100	NR	NR	NR	NR	Vegetarians/vegans: 15 Omnivores: 103	EAT‐26
Lindeman (2002), Finland^b^	239	29.0	Female = 100	NR	NR	NR	NR	Vegetarians/vegans: 42 omnivores: 197	EAT‐26
McLean et al. (2021), Australia, Canada, UK, USA	228	NR	Female = 51.8 male = 48.2	NR	NR	25.3	NR	Vegans: 110 omnivores: 118	EAT‐26, EDE‐Q
McLean et al. (2022), Australia^c^	1271	29.9	Female = 82.0	Australian (494)	Least SES disadvantage (58.9%)	24.7	63.6	Vegetarians: 278 vegans: 580 omnivores: 413	EDE‐Q
McLean et al. (2023), Australia^c^	1271	29.9	Female = 82.0	Australian (494)	Least SES disadvantage (58.9%)	24.7	63.6	Vegetarians: 278 vegans: 580 omnivores: 413	EAT‐26
Norwood et al. (2019), Australia	393	29.4	Female = 83.0 male = 17.0	Caucasian (310) Asian (43) Other (39)	NR	23.0	28.3	Vegetarians: 48 vegans: 128 omnivores: 101	EDI‐3
Paslakis et al. (2020), Germany	2449	49.6	Female = 53.5 male = 46.5	NR	Income groups Euro/month: 0 to < 1000 (23.9%) 1000 to < 2500 (62.2%) ≥ 2500 (10.7%)	25.9	23.4	Vegetarians/vegans: 133 omnivores: 2316	EDE‐Q8
Perry et al. (2001), USA	4510	14.9	Female = 49.8 male = 50.2	Caucasian (2302) African American (902) Aian American (911) Hispanic (275) native American (166) mixed/other (185)	NR	NR	0	Vegetarians/vegans: 94 omnivores: 4416	Self‐reported diagnosis
Sieke et al. (2021), USA	1585	20.9	Female = 60.0	Caucasian (1173) Asian (156) Hispanic (89) African American (77) other (90)	NR	NR	100	Vegetarians/vegans:136 omnivores: 1398	EDE‐Q
Timko et al. (2012), USA	486	25.1	Female = 77.0	Caucasian (388) Asian/Pacific Islanders (40) African American (23) Hispanic/Latino (15) Biracial (11) other (7)	NR	24.0	NR	Vegetarians: 111 vegans: 35 omnivores: 265	EAT‐26
Zickgraf et al. (2020), USA	9910	23.4	Cisgender women = 54.3 cisgender men = 44.4 transgender men = 0.2 transgender women = 0.2 genderqueer/gender nonconforming = 0.7 other = 0.2	Non‐Hispanic White (6639) Non‐Hispanic Black/African American (535) Hispanic/Latinx (931) Asian (1318) other (505)	NR	23.2	100	Vegetarians/vegans: 968 omnivores: 8926	S‐EDE‐Q

*Note:* All studies are cross‐sectional.

Abbreviations: BMI, body mass index; EAT, Eating Attitudes Test; ED, eating disorder; EDE‐Q, Eating Disorder Examination‐Questionnaire; EDI, Eating Disorder Inventory; NR, not reported; SCOFF, Sick‐Control‐Fat‐Food questionnaire; S‐EDE‐Q, Short‐Eating Disorder Examination‐Questionnaire; SES, socioeconomic status.

^a^ Vegan participants in Heiss et al. (2019) overlapped with those included in Heiss, Hormes, and Timko (2017); therefore, only vegetarian participants from the 2019 study were retained to prevent double counting.

^b^ To avoid double counting, only the adolescent sample from Lindeman et al. (2000) was included, as the adult participants were subsequently reported in Lindeman (2002).

^c^ These studies analyzed different scales measuring eating disorder symptoms within the same study sample.

Regarding dietary exposure, studies included data from vegetarians (Gwioździk et al. 2022), vegans (Heiss, Coffino, and Hormes 2017; McLean et al. 2021), vegetarians and vegans combined (Białek‐Dratwa et al. 2024; Çiçekoğlu and Tunçay 2018; Dorard and Mathieu 2021; Forestell et al. 2012; Gorgoni et al. 2026; Köse et al. 2021; Lindeman 2002; Lindeman et al. 2000; Paslakis et al. 2020; Perry et al. 2001; Sieke et al. 2021; Zickgraf et al. 2020), and vegetarians and vegans separately (Barnett et al. 2016; Coimbra and Ferreira 2021; Collins and Quinton 2020; Hanras et al. 2022; Heiss et al. 2019; McLean et al. 2022, 2023; Norwood et al. 2019; Timko et al. 2012). Only four studies (Dorard and Mathieu 2021; Forestell et al. 2012; McLean et al. 2022; Timko et al. 2012) reported the mean duration of adherence to VVD, which ranged from 5 to 10 years. Two studies analyzed ED symptoms according to the motivation for following such diets (Sieke et al. 2021; Zickgraf et al. 2020), and one study provided sex‐stratified results (McLean et al. 2021).

Regarding the instruments to assess ED symptoms, 10 studies employed the Eating Attitudes Test (EAT) (Białek‐Dratwa et al. 2024; Çiçekoğlu and Tunçay 2018; Collins and Quinton 2020; Dorard and Mathieu 2021; Forestell et al. 2012; Hanras et al. 2022; Köse et al. 2021; Lindeman 2002; Lindeman et al. 2000; McLean et al. 2021, 2023; Timko et al. 2012), nine studies used the ED Examination Questionnaire (EDE‐Q) (Barnett et al. 2016; Coimbra and Ferreira 2021; Gorgoni et al. 2026; Heiss, Coffino, and Hormes 2017; Heiss et al. 2019; McLean et al. 2021, 2022; Paslakis et al. 2020; Sieke et al. 2021; Zickgraf et al. 2020), one study employed the Eating Disorder Inventory (EDI) (Norwood et al. 2019), one study applied the Eating Disorder Diagnostic Scale (Barnett et al. 2016), one study used the Sick‐Control‐One‐Fat‐Food questionnaire (SCOFF) (Gwioździk et al. 2022), and one study assessed ED status through self‐reported diagnosis (McLean et al. 2021; Perry et al. 2001). No studies employed observer‐rated instruments.

### 3.3 Narrative Synthesis of Study Findings

Overall, findings across studies were heterogeneous (Table S3). Ten studies reported higher ED symptom scores among individuals following VVDs compared with omnivorous diets (Köse et al. 2021; Lindeman 2002; Lindeman et al. 2000; McLean et al. 2021, 2022, 2023; Paslakis et al. 2020; Sieke et al. 2021; Timko et al. 2012; Zickgraf et al. 2020), 10 found no significant differences between dietary groups (Barnett et al. 2016; Białek‐Dratwa et al. 2024; Çiçekoğlu and Tunçay 2018; Coimbra and Ferreira 2021; Dorard and Mathieu 2021; Forestell et al. 2012; Gwioździk et al. 2022; Heiss et al. 2019; Norwood et al. 2019; Perry et al. 2001), and four reported lower ED symptom scores among individuals following VVDs (Collins and Quinton 2020; Gorgoni et al. 2026; Hanras et al. 2022; Heiss, Coffino, and Hormes 2017). In some studies, associations varied in magnitude and, occasionally, in direction according to the dietary pattern examined as exposure (i.e., vegetarian or vegan diets) (Barnett et al. 2016; Coimbra and Ferreira 2021; Collins and Quinton 2020; Hanras et al. 2022; Heiss, Coffino, and Hormes 2017; Heiss et al. 2019; Norwood et al. 2019; Timko et al. 2012) and the instrument used to assess ED symptoms (McLean et al. 2021). The limited number of studies examining motivations for dietary adherence also suggested that associations may differ according to the reasons underlying VVD adoption (Sieke et al. 2021; Zickgraf et al. 2020). One study found higher ED symptom levels among individuals following VVDs for weight‐related reasons (Zickgraf et al. 2020), whereas another found that weight‐ and health‐related motivations were not associated with higher ED symptom levels among vegetarians and vegans (Sieke et al. 2021; Zickgraf et al. 2020). Findings from studies reporting categorical data were also heterogeneous (Table S3). Two studies reported a higher proportion of participants exceeding ED screening thresholds among individuals following VVDs compared with omnivorous diets (Köse et al. 2021; McLean et al. 2022), one reported a lower proportion (Hanras et al. 2022), and six found no significant differences between dietary groups (Barnett et al. 2016; Białek‐Dratwa et al. 2024; Dorard and Mathieu 2021; Gwioździk et al. 2022; Perry et al. 2001; Sieke et al. 2021). A meta‐analysis of categorical outcomes was not conducted because outcome definitions varied substantially across studies, including self‐reported diagnoses and different instrument‐specific screening thresholds, precluding the standardization of effect estimates.

### 3.4 Methodological Quality of the Included Studies

According to the NIH Quality Assessment Tool, study scores ranged from four to seven points. The most frequently unmet criteria included formal sample size justification or power calculations, exposure assessed prior to outcome, sufficient timeframe, repeated exposure assessment, and blinding of outcome assessors (Table S4). However, some unmet criteria reflected limitations inherent to cross‐sectional designs (e.g., exposure assessed prior to outcome, sufficient timeframe, repeated exposure assessment) or the use of self‐reported outcomes (e.g., blinding of outcome assessors), rather than necessarily indicating poor overall methodological rigor.

### 3.5 Meta‐Analysis

The main meta‐analysis is displayed in Figure 2. No significant differences in ED symptom scores were observed between individuals following VVDs (*n* = 4448) and those following omnivorous diets (*n* = 20,775), with a pooled SMD of 0.07 (95% CI: −0.04 to 0.19; *I* ^2^ = 80.3%; prediction interval: −0.44 to 0.59; *n* = 23) in analyses including both adolescent and adult populations. Results were similar in analyses restricted to adult populations (SMD = 0.05; 95% CI: −0.07 to 0.16; *I* ^2^ = 79.7%; prediction interval: −0.41 to 0.50; *n* = 19; Figure S1).

**FIGURE 2:**
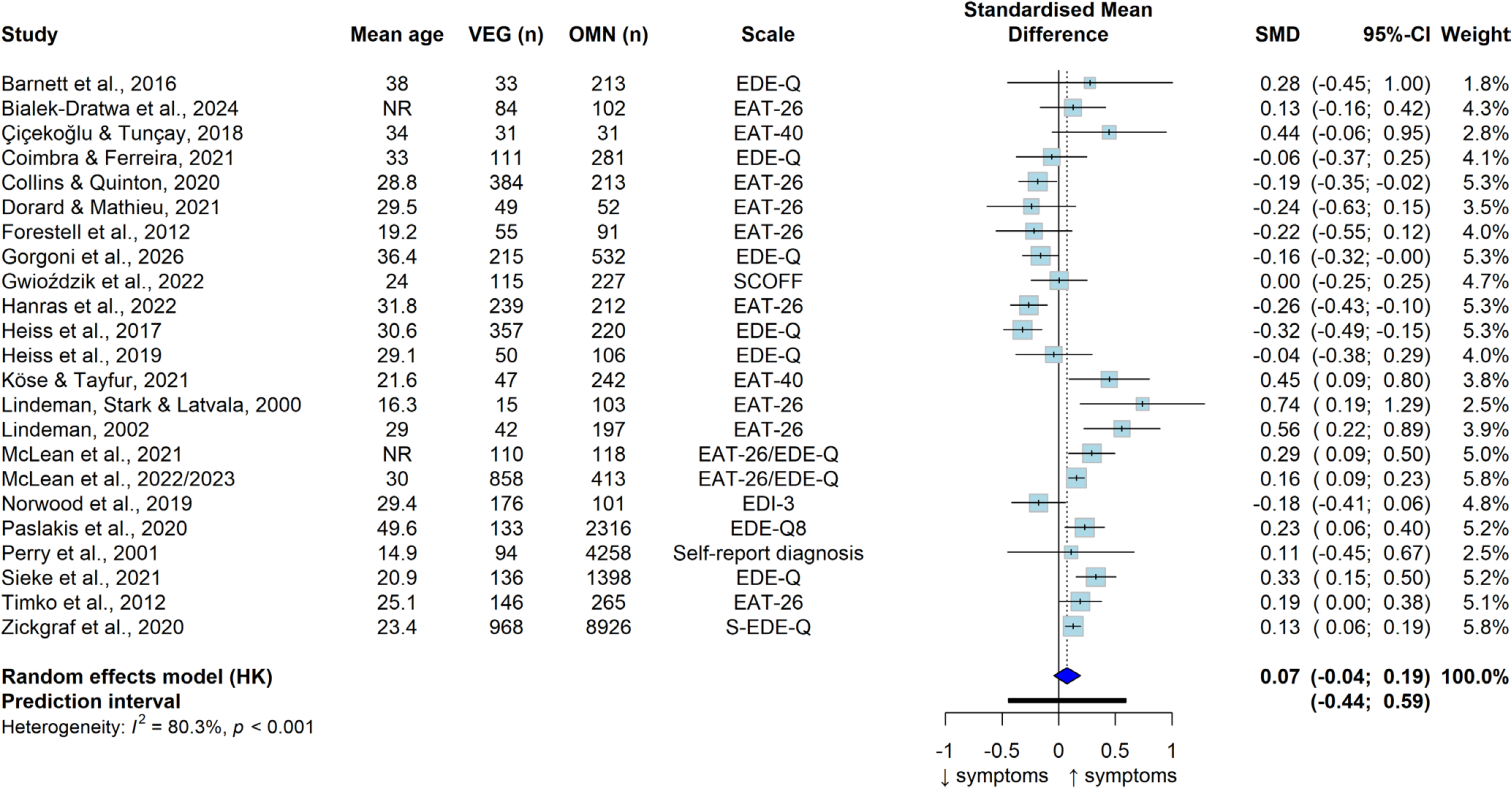
Pooled standardized mean differences in eating disorder symptoms comparing vegetarian and vegan with omnivorous diets in studies including adolescent and adult populations. CI, confidence interval; EAT, Eating Attitudes Test; EDE‐Q, Eating Disorder Examination‐Questionnaire; EDI, Eating Disorder Inventory; HK, Hartung‐Knapp; OMN, omnivorous; SCOFF, Sick‐Control‐Fat‐Food questionnaire; S‐EDE‐Q, Short‐Eating Disorder Examination‐Questionnaire; SMD, standardized mean difference; VEG, vegetarians and vegans.

The results of the subgroup analyses are displayed in Figure 3 and Table S5. No significant differences in ED symptoms were observed according to diet type (*p* = 0.524), measurement scale (*p* = 0.392), or geographic region (*p* = 0.633). Compared with omnivorous individuals, the pooled SMDs were 0.02 (95% CI: −0.09 to 0.14; *I* ^2^ = 40.3%; *n* = 10) for vegetarians, and −0.05 (95% CI: −0.29 to 0.20; *I* ^2^ = 86.7%; *n* = 10) for vegans. By measurement scale, the pooled SMDs were 0.15 (95% CI:–0.06 to 0.37; *I* ^2^ = 83.2%; *n* = 12) for the EAT, and 0.06 (95% CI: −0.09 to 0.20; *I* ^2^ = 80.1%; *n* = 10) for the EDE‐Q. Across geographic regions, pooled SMDs were 0.11 (95% CI: −0.17 to 0.38; *I* ^2^ = 82.1%; *n* = 8) for European countries, 0.04 (95% CI: −0.16 to 0.24; *I* ^2^ = 80.5%; *n* = 8) for North America (USA only), and −0.05 (95% CI: −0.56 to 0.45; *I* ^2^ = 89.5%; *n* = 4) for Oceania (Australia only). No substantial differences were observed in subgroup analyses restricted to adult populations (Table S5). Due to the small number of studies examining ED symptoms within specific subgroups (i.e., Asia, EDI, SCOFF), stratified analyses were not conducted. In addition, only one study reported sex‐stratified results, and none examined age‐specific results.

**FIGURE 3:**
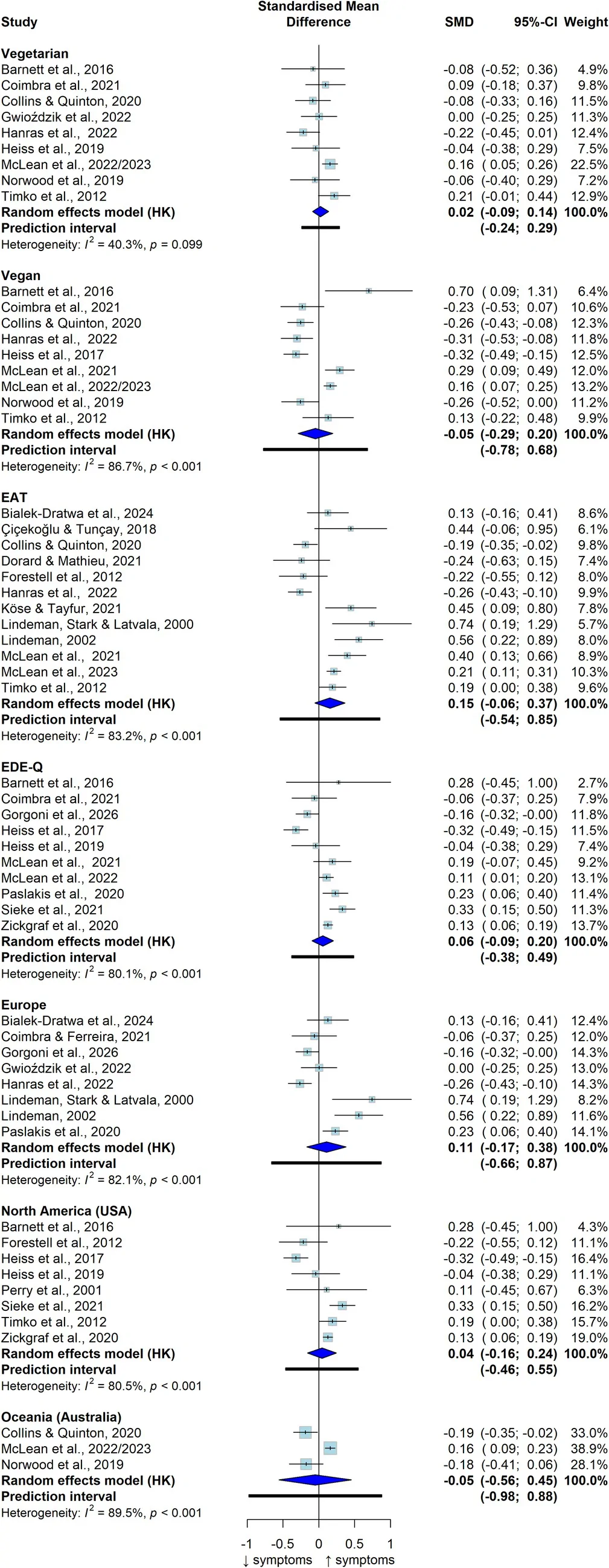
Pooled standardized mean differences in eating disorder symptoms comparing vegetarian and vegan with omnivorous diets by subgroups in studies including adolescent and adult populations. CI, confidence interval; EAT, Eating Attitudes Test; EDE‐Q, Eating Disorder Examination‐Questionnaire; HK, Hartung‐Knapp; OMN, omnivorous; SMD, standardized mean difference; VEG, vegetarians and vegans.

Meta‐regression analyses according to participant and study characteristics are displayed in (Table S6) and (Figure S2,S3). The proportion of female participants was significantly associated with the pooled SMD in analyses restricted to adult populations (*β* = −0.008, *p* = 0.028; *R* ^2^ = 20.6%). Specifically, studies including a higher proportion of female participants tended to report lower ED symptom scores among individuals following VVDs compared with omnivorous diets (Figure S3B). No other participant or study characteristics (age, total sample size, BMI, underweight status, educational level, and publication year) were significantly related to the pooled effect size (*p* > 0.05). However, underweight status explained a proportion of the between‐study heterogeneity (*R* ^2^ = 28.9%) and showed a nonsignificant positive association with the pooled effect estimate (*β* = 0.062, *p* = 0.095), suggesting a tendency toward higher ED symptom scores among adults following VVDs in studies with a greater proportion of underweight participants (Figure S3E). A trend was also observed for publication year, which explained a modest proportion of the between‐study heterogeneity (*R* ^2^ = 13.4%) and showed a nonsignificant inverse association with the pooled effect estimate (*β* = −0.017, *p* = 0.057), suggesting a tendency for more recently published studies to report smaller effect sizes than earlier studies (Figure S2F).

The results of the sensitivity analysis are presented in Table S7. The pooled SMDs remained stable when each study was removed sequentially, and no substantial variations in between‐study heterogeneity were observed. According to Egger's regression test and visual inspection of the funnel plot, no evidence of publication bias was detected (*p* = 0.758; Figure S4).

## 4 Discussion

To the best of our knowledge, this is the first meta‐analysis is the first to quantify the associations between ED symptoms and VVDs in relation to omnivorous diets. Our findings suggest that individuals adhering to VVDs do not report significantly higher levels of ED symptoms than those following omnivorous diets. These results are based on study samples predominantly composed by female sex and by young and middle‐aged adults. Subgroup analyses revealed no significant differences according to dietary pattern type (vegetarian or vegan), geographic region (Europe, North America, or Oceania), or scale used to assess ED symptoms (EAT or EDE‐Q). In addition, no significant associations were found between the pooled effect size and some participant and study characteristics assessed in meta‐regressions, including age, total sample size, BMI, educational level, and publication year. However, the pooled effect was moderated by sex in analyses restricted to adult populations. Studies with a higher proportion of females showed lower ED symptom scores among vegetarian or vegan adults compared to omnivores. Findings should be interpreted with caution given the high heterogeneity and lack of longitudinal data.

Previous reviews were limited to qualitative syntheses and yielded heterogeneous, inconclusive findings regarding the association between vegetarian diets and EDs (Mathieu et al. 2023; McLean et al. 2022a; Sergentanis et al. 2020). The discrepancies with previous findings may be partly explained by important methodological and conceptual differences between studies, as well as differences in the age range of the populations examined. Definitions of vegetarian dietary patterns have varied substantially, with several studies grouping heterogeneous dietary regimens under vegetarian categories, including semi‐vegetarian, flexitarian, or pescatarian diets that allow occasional meat or fish consumption (Mathieu et al. 2023; Sergentanis et al. 2020). This distinction may be relevant, as semi‐vegetarian patterns have been associated with greater eating‐related psychopathology than strict VVDs (Heiss, Hormes, and Timko 2017). In addition, previous reviews have examined a broad range of outcomes, including clinical diagnosis of EDs, ED symptoms, disordered eating behaviors, and orthorexia nervosa (Mathieu et al. 2023; McLean et al. 2022a; Sergentanis et al. 2020). As these outcomes represent related but distinct constructs, direct comparisons across reviews may be challenging. Notably, some reviews have reported more consistent associations between VVDs and orthorexia tendencies than with global ED psychopathology or ED symptoms (Mathieu et al. 2023; McLean et al. 2022a), while a recent meta‐analysis also reported a positive association between VVDs and symptoms of orthorexia nervosa (Díaz‐Goñi et al. 2025). Together, these considerations suggest that differences in exposure definitions and outcome assessment should be carefully considered when interpreting the available evidence.

In this context, the present meta‐analysis provides quantitative evidence indicating that adherence to strict VVDs is not associated with higher levels of ED symptoms compared with omnivorous diets. The absence of an overall association may partly reflect the heterogeneity of motivations underlying adherence to VVDs. In some populations, these dietary patterns are primarily adopted for health, ethical, environmental, or animal welfare concerns (Kim et al. 2022; Miki et al. 2020; Rosenfeld 2018; Rosenfeld and Burrow 2017; Ruby 2012) rather than weight‐control motives, which may be more strongly linked to ED psychopathology (Bardone‐Cone et al. 2012). However, the potential influence of these motivations on ED symptomatology remains unclear because they were not consistently assessed across studies. From a public health and clinical perspective, these findings suggest that adherence to VVDs should not be uniformly interpreted as an indicator of increased ED risk. Considering the growing popularity of these dietary patterns, clinicians and researchers should consider the broader context underlying dietary choices rather than focusing exclusively on the dietary pattern itself. Greater attention may be warranted when restrictive dietary practices coexist with weight‐control motivations or clinically relevant ED symptoms.

This systematic review further extends the available evidence through subgroup analyses and meta‐regressions exploring potential sources of heterogeneity. The higher heterogeneity observed in studies including vegan participants compared with vegetarian participants may partly reflect the smaller number of participants in the vegan subgroup and could also be related to variability in motivations for dietary adherence. In addition, meta‐regression analyses indicated that differences in the proportion of female participants and individuals classified as underweight across studies may contribute to the observed between‐study heterogeneity. Specifically, studies including a higher proportion of females tended to report lower ED symptom scores among individuals adhering to VVDs compared with omnivorous diets. The finding should be interpreted cautiously and viewed as a gradual trend across studies rather than a distinct association among studies with predominantly female samples. It may suggest that the meaning and motivations underlying adherence to VVDs differ across populations. In samples with a higher proportion of female, VVDs may be more frequently adopted for ethical, environmental, or health‐related reasons rather than weight‐control purposes (MacInnis and Hodson 2017; Rosenfeld 2020), potentially weakening their association with ED symptoms. Alternatively, this finding may reflect other unmeasured sociocultural characteristics of the included samples. Future studies should examine whether motivations for dietary adherence explain sex‐related differences in the association between VVDs and ED symptoms. From a public health perspective, these findings suggest that the assessment of ED risk among individuals adhering to VVDs should consider the motivations underlying dietary choices rather than focusing exclusively on the dietary pattern itself.

A nonsignificant trend was also observed for the proportion of underweight participants, with studies including a higher proportion of these individuals tending to report higher ED symptom scores among those adhering to VVDs compared with omnivorous diets. Although these findings should be interpreted cautiously, they may suggest that VVDs can, in some individuals, provide a socially acceptable framework for dietary restrictions, particularly among those with greater weight concerns or preexisting disordered eating behaviors (Gilbody et al. 1999). A further trend was observed for publication year, with more recently published studies tending to report smaller effect sizes than earlier studies. Although this association did not reach statistical significance, it may reflect temporal and sociocultural changes in the acceptance and motivations underlying VVDs. However, this finding should be interpreted cautiously because publication year may also capture differences in study populations and methodologies.

The substantial between‐study heterogeneity also suggests the influence of methodological variability. One possible explanation relates to the questionnaires used to assess ED symptoms, as most instruments were developed for the general population and may not fully capture eating‐related behaviors among individuals following specific dietary patterns such as VVDs. Concerns have been raised regarding the psychometric performance of some commonly used ED instruments in vegetarian and vegan populations, as certain items may capture normative dietary practices rather than ED psychopathology (Mathieu et al. 2023; McLean et al. 2022a). For example, instruments such as the EDE‐Q include restraint‐related items that may not clearly distinguish between psychopathological eating restraint and food restrictions arising from ethical, environmental, or health‐related dietary choices. Consequently, widely used tools may not adequately reflect the experiences of individuals adhering to VVDs (McLean et al. 2022a). In response to these limitations, assessment instruments specifically tailored to vegetarian and vegan populations are currently being developed and validated (McLean et al. 2024).

Contextual and cultural factors may also contribute to the observed heterogeneity. Although no statistically significant geographical differences were detected in the present study, the analyses were restricted to studies conducted in North America (USA), Europe (France, Germany, Poland, and Portugal), and Oceania (Australia), limiting the representation of other world regions. Previous research has documented substantial cross‐cultural variation in the occurrence and clinical presentation of EDs (Nobakht and Dezhkam 2000; Song et al. 2023). In some non‐Western contexts, restrictive eating behaviors may be framed in culturally sanctioned terms and may not be accompanied by an explicit fear of weight gain, despite underlying weight‐related concerns (Rieger et al. 2001). Cultural differences may also influence the social meaning and motivations underlying adherence to VVDs. Given that most included studies were conducted in Western populations, our findings may not be generalizable to cultural contexts in which plant‐based diets are more prevalent, socially normative, or rooted in long‐standing religious and cultural traditions. Future studies from underrepresented regions, including parts of Asia, Africa, and Latin America, are needed to better understand whether the association between VVDs and ED symptoms differs across cultural contexts.

### 4.1 Limitations and Strengths

Some limitations should be noted. First, the available evidence reported only cross‐sectional studies and prevented consistent conclusions about whether VVDs lead to the development of ED symptoms. Second, our analyses revealed substantial between‐study heterogeneity in the main pooled estimate, partially attributable to differences in diet type, geographic region, the instruments used to assess ED symptoms, and characteristics of study samples (percentages of females and underweight status). Third, the small number of studies with age‐ and sex‐stratified data prevented us from examining potential differences in these population characteristics. Fourth, data and analyses of VVD adherence, specifically regarding duration, motivation, and quality, are lacking in all or most studies, preventing key analyses that could help explain the observed associations. Moreover, most studies classified VVDs based on self‐reported adherence rather than objectively assessed dietary intake, which may have resulted in exposure misclassification. Fifth, most studies did not report sociodemographic characteristics, such as ethnicity, race, gender, and socioeconomic status. This lack of information limits the ability to evaluate the representativeness and diversity of the samples, as well as the generalizability of the findings to broader populations. Sixth, all included studies were conducted in nonclinical populations. Therefore, our findings are primarily relevant to prevention, screening, and public health contexts and should not be extrapolated to individuals with diagnosed EDs or those undergoing treatment or recovery. Consequently, the present review cannot determine whether adherence to vegetarian or vegan diets may influence the course, treatment, or recovery of EDs. Finally, most studies did not provide information on potentially influential covariates, such as physical activity, socioeconomic status, weight loss, and the use of social networks. This limited our ability to perform adjusted analyses for these factors.

However, several important strengths distinguish our work. First, to the best of our knowledge, this is the first meta‐analysis to quantify the association between VVDs and omnivorous diets. Second, this approach enabled the implementation of a random‐effects meta‐analytic model using the Hartung–Knapp–Sidik–Jonkman method, which has been shown to yield more conservative and robust CIs in comparison to traditional methods, particularly in the presence of heterogeneity and a limited number of studies (Inthout et al. 2014). Third, subgroup analyses were conducted to explore potential sources of heterogeneity and to assess variations in effect size according to key variables relevant to the study association. Fourth, meta‐regression analyses were performed to evaluate the potential influence of relevant confounders on the observed associations. Fifth, prediction intervals were calculated to estimate the range of actual effects that could be expected in future studies, providing a more realistic assessment of the variability between studies and the certainty of the results. Finally, only studies that explicitly defined vegetarian diets as strict—excluding any type of meat—were included (e.g., excluding semi‐vegetarians, flexitarians, and pescatarians), to ensure clarity in the research question and to provide more accurate and interpretable results.

### 4.2 Future Recommendations

Longitudinal research remains essential to clarify the directionality and causal mechanisms linking diet with ED. In our study, individuals adhering to VVDs are not more likely to report ED symptoms than those following omnivorous diets. However, the underlying motivations for adopting VVDs may play a crucial role in this relationship. Across the included evidence, one study (Zickgraf et al. 2020) reported that adopting VVDs for weight‐related reasons was linked to greater symptom levels compared with non–weight‐related reasons. In turn, adjusted models from a recent study found that motives such as liking and health were negatively associated with disordered eating attitudes, whereas other motives such as visual appeal, social image, weight control, and affect regulation were positively associated with higher levels of these attitudes (Mazzolani et al. 2023). Among the general population, motivations driven by ethical or health considerations could differ in psychological meaning and behavioral implications from those influenced by perceived social expectations or body image concerns (Mazzolani et al. 2023; Sharpe et al. 2018). This highlights the importance of distinguishing the reasons why individuals adopt VVDs rather than assuming these dietary patterns are inherently linked to psychopathology.

Discouraging VVDs among individuals whose dietary choices are closely tied to their personal identity or values may therefore be counterproductive, potentially reinforcing distress rather than supporting healthier relationships with food (McLean et al. 2022b). In this context, previous evidence from qualitative research has proposed five key indicators to help identify potentially pathological forms of VVDs adherence, including the timing of dietary change relative to the onset of disordered eating, the motivations for dietary adherence, fear‐driven reactions, flexibility within vegetarian or vegan practices, and the potential use of dietary choices to conceal ED behaviors (McLean et al. 2025).

Future research should adopt longitudinal designs and include a broader range of age groups. Given that most available evidence is derived from young and middle‐aged adults, further research in adolescent and older populations is warranted. Research should also prioritize populations from understudied geographical regions, cultural contexts, and ethnic and racial backgrounds. In addition, studies conducted in clinical populations, including individuals with diagnosed EDs and those undergoing treatment or recovery, are needed to determine whether adherence to VVDs influences treatment outcomes, symptom progression, or recovery trajectories. It will also be essential to examine whether diet quality within vegetarian and vegan patterns—ranging from highly processed to whole‐food plant‐based approaches—modifies associations with ED outcomes, given that nutritional adequacy and food quality may be key determinants of mental health trajectories (Bizzozero‐Peroni et al. 2025). In addition, the duration and flexibility for VVD adherence should be considered, as these factors may further influence psychological outcomes. Finally, future studies should assess the role of additional factors such as gender, weight loss, social media exposure, physical activity, tobacco smoking, and alcohol consumption, which may influence both dietary motivations and eating‐related vulnerability.

## 5 Conclusion

Across the available evidence, no significant differences in ED symptoms were observed between individuals following VVDs and those following omnivorous diets. These findings suggest that dietary patterns alone may not necessarily be associated with an increased risk of ED. However, the results should be interpreted with caution given the substantial between‐study heterogeneity and the exclusively cross‐sectional nature of the available evidence, which precludes conclusions regarding causality and may affect the robustness of the findings. Moreover, the available evidence was predominantly derived from young and middle‐aged adults, highlighting the need for additional research in adolescent and older populations. Future longitudinal studies across diverse geographical and cultural contexts—and reporting stratified analyses by age, sex, ethnicity/race, diet quality, and flexibility, motivation, and duration of VVD adherence—are essential to clarify the directionality and underlying mechanisms of these associations. Such evidence could inform early screening and the development of personalized prevention strategies in public health and community settings.

## Author Contributions

**Arthur Eumann Mesas:** conceptualization, investigation, methodology, writing – original draft, visualization, project administration, writing – review and editing, supervision. **Valentina Díaz‐Goñi:** conceptualization, investigation, methodology, formal analysis, visualization, writing – original draft, writing – review and editing. **Estela Jiménez‐López:** writing – original draft, writing – review and editing. **Bruno Bizzozero‐Peroni:** writing – review and editing, conceptualization, investigation, methodology, formal analysis, writing – original draft, visualization, validation, project administration, supervision. **Ana María Peraile‐Huerta:** validation, writing – review and editing. **Miriam Garrido‐Miguel:** validation, writing – review and editing. **Tomás Olivo‐Martins‐de‐Passos:** validation, writing – review and editing. **María Eugenia Visier‐Alfonso:** writing – original draft, writing – review and editing. **Fernando Peral‐Martínez:** validation, writing – review and editing.

## Lived Experience Involvement Statement

No specific efforts were undertaken to involve persons with lived experience in the study design or execution, or in the preparation of this manuscript.

## Funding

This work was supported by multiple funding sources. V.D.‐G. is supported by the Regional Government of Castilla‐La Mancha (2025‐CACT‐13334), and co‐funded by the European Union (grant code SBPLY/23/180225/000177), and the National Agency for Research and Innovation, Uruguay (POS_EXT_2023_1_175630). T.O.‐M.‐d‐P. is hired (2025‐CACT‐13335) under a project funded by the Ministry of Health, Consumer Affairs, and Social Welfare, Spain (grant number 147806). F.P.‐M. holds a pre‐doctoral contract (grant code FPU24/01605) funded by the Ministry of Science, Innovation and Universities, Spain. B.B.‐P. is supported by a grant from the University of Castilla‐La Mancha co‐financed by the European Social Fund (2024‐UNIVERS‐12849). The research groups involved in this study received financial support from the Ministry of Finance, Spain, co‐financed by the European Union FEDER Funds and the Castilla‐La Mancha regional government (2022‐GRIN‐34427, 2025‐GRIN‐38383, and 2025‐GRIN‐38304). Additional support was provided by European Union funding programs, including the European Regional Development Fund (FEDER) and NextGenerationEU, and the Ministry of Economy and Competitiveness, Spain (RD21/0016/0025). The funders did not play a role in the design of the study, the collection or analysis of the data, or the publication decisions.

## Disclosure

No generative AI tools were used in the preparation of this manuscript.

## Ethics Statement

The authors have nothing to report.

## Consent

The authors have nothing to report.

## Conflicts of Interest

The authors declare no conflicts of interest.

## Supporting information


**Table S1:** Detailed information on search strategies.
**Table S2:** Studies excluded after full‐text assessment.
**Table S3:** Effect sizes, main results, and covariate adjustments of included studies.
**Table S4:** Methodological quality of included studies.
**Table S5:** Subgroup analyses for the cross‐sectional associations between vegetarian and vegan versus omnivorous diets and eating disorder symptoms.
**Table S6:** Meta‐regression analyses for the cross‐sectional associations between vegetarian and vegan versus omnivorous diets and eating disorder symptoms.
**Table S7:** Sensitivity analyses excluding studies one by one.
**Figure S1:** Pooled standardized mean differences in eating disorder symptoms comparing vegetarian and vegan with omnivorous diets in studies including adult populations.
**Figure S2:** Meta‐regression models by age (A), females (B), total sample size (C), Body Mass Index (D), underweight status (E), and publication year (F) for studies including both adolescent and adult populations.
**Figure S3:** Meta‐regression models by age (A), females (B), total sample size (C), Body Mass Index (D), underweight status (E), educational level (F) and publication year (G) for studies restricted to adult populations. Notes: (A) Mean age (years); (B) Percentage of females; (C) total sample size; (D) Body Mass Index (mean, kg/m2); (E) Percentage of underweight status (BMI < 18 kg/m2); (F) Percentage of high educational level (university degree); and (G) Publication year.
**Figure S4:** Funnel plot of publication bias.

## Data Availability

The data that support the findings of this study are available from the corresponding author upon reasonable request.
