# Supplementary material for: Are Vegetarian and Vegan Diets Associated With Eating Disorder Symptoms? A Systematic Review and Meta‐Analysis
Source: Int J Eat Disord. 2026 Jul 7;59(11):2347–63. doi: 10.1002/eat.70158 (PMC13726445; doi:10.1002/eat.70158)
Supplement: Supplementary file 1 [file EAT-59-2347-s001.docx]

**Supplementary Material**

**Are vegetarian and vegan diets associated with eating disorder symptoms? A systematic review and meta-analysis**

**Supplementary Table 1.** Detailed information on search strategies.

| **Information source** | **Method** |
| --- | --- |
| Databases^a^: PubMed^b^, Scopus^c^, Cochrane Library^c^, PsycINFO^c^ and Web of Science^c^ | (vegetarian* OR vegan* OR "plant-based" OR "plant based" OR "vegetable diet*" OR "plant-rich diet*" OR "meat-free" OR "meat free" OR "meat avoidance" OR "meat-avoid*" OR "nonmeat eat*" OR Adventist) AND ("eating disorder*" OR "disordered eating" OR "eating symptom*" OR "eating attitude*" OR "eating problem*" OR anorexi* OR bulimi* OR hyperphagia OR binge* OR overeating OR "avoidant food" OR "avoidant eating disorder" OR "Avoidant Restrictive Food Intake Disorder" OR "Avoidant/Restrictive Food Intake Disorder" OR "restrictive eating disorder" OR "selective eating disorder" OR "food avoidance disorder" OR "feeding disorder*" OR "compensatory behavio*" OR "extreme diet*" OR "cognitive restraint" OR "patholog* eating" OR "eating patholog*" OR SCOFF) |
| Online resources and browsing | Portal (URL): Google Scholar ([https://scholar.google.com](https://scholar.google.com/)). Search terms: (vegetarian OR vegan) AND (“disordered eating OR “eating disorder”). The first 100 records (n=10 pages) were selected and screened. |
| Citation searching | Relevant systematic reviews were kept separate to screen their included studies^1-3^. |

^a^Data were retained in Research Information Systems (RIS) or PubMed formats for each database. ^b^ Search strategy restricted to Title/Abstract. ^c^ Search strategy restricted to Title/Abstract/Keywords.

References cited in this table:

^1^ Mathieu S, Hanras E, Dorard G. Associations between vegetarianism, body mass index, and eating disorders/disordered eating behaviours: a systematic review of literature. Int J Food Sci Nutr 2023; 74: 424–462.

^2^ McLean CP, Kulkarni J, Sharp G. Disordered eating and the meat-avoidance spectrum: a systematic review and clinical implications. Eating and Weight Disorders 2022; 27: 2347–2375.

^3^ Sergentanis TN, Chelmi ME, Liampas A, Yfanti CM, Panagouli E, Vlachopapadopoulou E, Michalacos S, Bacopoulou F, Psaltopoulou T, Tsitsika A. Vegetarian Diets and Eating Disorders in Adolescents and Young Adults: A Systematic Review. Children (Basel). 2020;8(1):12.

**Supplementary Table 2.** Studies excluded after full-text assessment.

**Wrong population (n=5)**

- Bardone-Cone AM, Fitzsimmons-Craft EE, Harney MB, Maldonado CR, Lawson MA, Smith R, Robinson DP. The inter-relationships between vegetarianism and eating disorders among females. J Acad Nutr Diet. 2012;112(8):1247-52. doi: 10.1016/j.jand.2012.05.007.
- Fan J, Nagata JM, Cuccolo K, Ganson KT. Associations between dieting practices and eating disorder attitudes and behaviors: Results from the Canadian study of adolescent health behaviors. Eat Behav. 2024;54:101886. doi: 10.1016/j.eatbeh.2024.101886.

**Wrong exposure (n=12)**

- Barrack MT, West J, Christopher M, Pham-Vera AM. Disordered Eating Among a Diverse Sample of First-Year College Students. J Am Coll Nutr. 2019;38(2):141-148. doi: 10.1080/07315724.2018.1487345.
- Baş M, Karabudak E, Kiziltan G. Vegetarianism and eating disorders: association between eating attitudes and other psychological factors among Turkish adolescents. Appetite. 2005;44(3):309-15. doi: 10.1016/j.appet.2005.02.002.
- Coimbra M, Ferreira C. Making the leap from healthy to disordered eating: the role of intuitive and inflexible eating attitudes in orthorexic behaviours among women. Eat Weight Disord. 2021;26(6):1793-1800. doi: 10.1007/s40519-020-00998-1.
- Douglas CC, Shahan M, Camel SP, Vogel S. Negative Body Image and Limited Nutrition Knowledge among Collegiate Dancers Signals Need for Screening and Support. J Dance Med Sci. 2025 Sep;29(3):161-169. doi: 10.1177/1089313X241297770. Epub 2024 Nov 19. PMID: 39560063.
- Fatima W, Ahmad LM. Prevalence of disordered eating attitudes among adolescent girls in Arar City, Kingdom of Saudi Arabia. Health Psychol Res. 2018;6(1):7444. doi: 10.4081/hpr.2018.7444.
- Fisak B Jr, Peterson RD, Tantleff-Dunn S, Molnar JM. Challenging previous conceptions of vegetarianism and eating disorders. Eat Weight Disord. 2006;11(4):195-200. doi: 10.1007/BF03327571.
- Gilbody SM, Kirk SF, Hill AJ. Vegetarianism in young women: another means of weight control? Int J Eat Disord. 1999;26(1):87-90. doi: 10.1002/(sici)1098-108x(199907)26:1<87::aid-eat11>3.0.co;2-m.
- Klopp SA, Heiss CJ, Smith HS. Self-reported vegetarianism may be a marker for college women at risk for disordered eating. J Am Diet Assoc. 2003;103(6):745-7. doi: 10.1053/jada.2003.50139.
- Michalak J, Zhang XC, Jacobi F. Vegetarian diet and mental disorders: results from a representative community survey. Int J Behav Nutr Phys Act. 2012;9:67. doi: 10.1186/1479-5868-9-67.
- Plichta M, Jezewska-Zychowicz M. Orthorexic Tendency and Eating Disorders Symptoms in Polish Students: Examining Differences in Eating Behaviors. Nutrients. 2020;12(1):218. doi: 10.3390/nu12010218.
- Robinson-O'Brien R, Perry CL, Wall MM, Story M, Neumark-Sztainer D. Adolescent and young adult vegetarianism: better dietary intake and weight outcomes but increased risk of disordered eating behaviors. J Am Diet Assoc. 2009;109(4):648-55. doi: 10.1016/j.jada.2008.12.014.
- Salvia MG, Onteeru M, Lipson SK, Quatromoni PA. Adopting vegetarian and vegan eating patterns: Associations with disordered eating behaviors among young adult college students. Eat Behav. 2025;57:101967. doi: 10.1016/j.eatbeh.2025.101967.
- Trautmann J, Rau SI, Wilson MA, Walters C. Vegetarian students in their first year of college: Are they at risk for restrictive or disordered eating behaviors? College Student Journal. 2008;42(2):340-347.

**Wrong outcome (n=11)**

- Albery IP, Smith R, Frings D, Spada M. Patterns of implicit and explicit identity as a vegan or vegetarian in predicting healthy orthorexia and orthorexia nervosa. Eat Weight Disord. 2025 Mar 18;30(1):27. doi: 10.1007/s40519-025-01734-3. PMID: 40100515; PMCID: PMC11920311.
- Bataineh MF, Alhebshi SH, Al-Nawaiseh AM, Jarrar AH, Hamad MI, Al Dhaheri AS, Cheikh Ismail L, Ali HI. Validity and reliability of the Arabic orthorexia nervosa inventory scale in young adults: a validation study. Eat Weight Disord. 2026 Mar 6;31(1):18. doi: 10.1007/s40519-026-01838-4. PMID: 41792404; PMCID: PMC12982198.
- Brinkman BG, Khan A, Edner B, Rosén LA. Self-objectification, feminist activism and conformity to feminine norms among female vegetarians, semi-vegetarians, and non-vegetarians. Eat Behav. 2014;15(1):171-4. doi: 10.1016/j.eatbeh.2013.11.006.
- Brytek-Matera A. Restrained Eating and Vegan, Vegetarian and Omnivore Dietary Intakes. Nutrients. 2020;12(7):2133. doi: 10.3390/nu12072133.
- Brytek-Matera A, Czepczor-Bernat K, Jurzak H, Kornacka M, Kołodziejczyk N. Strict health-oriented eating patterns (orthorexic eating behaviours) and their connection with a vegetarian and vegan diet. Eat Weight Disord. 2019;24(3):441-452. doi: 10.1007/s40519-018-0563-5.
- Reuber H, Muschalla B. Dietary identity and embitterment among vegans, vegetarians and omnivores. Health Psychol Behav Med. 2022;10(1):1038-1055. doi: 10.1080/21642850.2022.2134870.
- Martins Rodrigues CJ, Galvão OF, Albuquerque LC. Analysis of Inflexibility and Eating Disorders According to the Theory of Control by Justifications and Immediate Consequences (TJC). Nutrients. 2026 Mar 23;18(6):1011. doi: 10.3390/nu18061011. PMID: 41901186; PMCID: PMC13028811.
- Saintila J, Calizaya-Milla YE, Javier-Aliaga DJ. Knowledge of Vegetarian and Nonvegetarian Peruvian Dietitians about Vegetarianism at Different Stages of Life. Nutr Metab Insights. 2021;14. doi: 10.1177/1178638821997123.
- Sandri E, Sguanci M, Cantín Larumbe E, Cerdá Olmedo G, Werner LU, Piredda M, Mancin S. Plant-Based Diets versus the Mediterranean Dietary Pattern and Their Socio-Demographic Determinants in the Spanish Population: Influence on Health and Lifestyle Habits. Nutrients. 2024;16(9):1278. doi: 10.3390/nu16091278.
- Webb JB, Tran T, Bauert L, Pringle S. Body- and Fitness-Related Shame Helps Explain the Association Between Internalized Weight Bias and Orthorexia Symptoms Among North American Female Yoga Professionals. J Acad Nutr Diet. 2025 Dec;125(12):1864-1876. doi: 10.1016/j.jand.2025.04.013. Epub 2025 Apr 28. PMID: 40306434.
- Zuromski KL, Witte TK, Smith AR, Goodwin N, Bodell LP, Bartlett M, Siegfried N. Increased prevalence of vegetarianism among women with eating pathology. Eat Behav. 2015;19:24-7. doi: 10.1016/j.eatbeh.2015.06.017.

**Wrong study design (n=1)**

- McLean CP, de Boer K, Bray M. "If There Are Restrictions Within the Restrictions, That's When You Can Probably Get Concerned": Key Indicators for Untying Vegetarianism and Veganism From Eating Disorder Pathology. Int J Eat Disord. 2025 Sep;58(9):1723-1731. doi: 10.1002/eat.24475. Epub 2025 May 23. PMID: 40406828; PMCID: PMC12423574.

**No study association (n=4)**

- Brytek-Matera A, Onieva-Zafra MD, Parra-Fernández ML, Staniszewska A, Modrzejewska J, Fernández-Martínez E. Evaluation of Orthorexia Nervosa and Symptomatology Associated with Eating Disorders among European University Students: A Multicentre Cross-Sectional Study. Nutrients. 2020;12(12):3716. doi: 10.3390/nu12123716.
- Heiss S, Timko CA, Hormes JM. Confirmatory factor analysis of the EDE-Q in vegans and omnivores: Support for the brief three factor model. Eat Behav. 2020;39:101447. doi: 10.1016/j.eatbeh.2020.101447.
- Lindeman M, Stark K. Pleasure, pursuit of health or negotiation of identity? Personality correlates of food choice motives among young and middle-aged women. Appetite. 1999;33(1):141-61. doi: 10.1006/appe.1999.0241.
- McLean JA, Barr SI. Cognitive dietary restraint is associated with eating behaviors, lifestyle practices, personality characteristics and menstrual irregularity in college women. Appetite. 2003;40(2):185-92. doi: 10.1016/s0195-6663(02)00125-3.

**Supplementary Table 3.** Effect sizes, main results, and covariate adjustments of included studies.

| ***References*** | ***Measure of ED symptoms*** | ***Reported statistic*** | ***Vegetarian*** | ***Vegan*** | ***Omnivores*** |
| --- | --- | --- | --- | --- | --- |
| *Barnett et al. (2016)* | EDE-Q | Mean ± SD | 1.28±1.2 | 2.16±1.67 | 1.37±1.10 |
|  | EDDS | N events (%), sub/full threshold | 1 (4.5) | 2 (18.2) | 18 (9.5) |
| *Białek-Dratwa et al. (2024)* | EAT-26 | Median (CI) | 9.5 (7.6, 11.4) | | 8.0 (6.8, 9.2) |
|  |  | N events (%), cutoff ≥20 | 20 (23.8) | | 15 (14.7) |
| *Çiçekoğlu & Tunçay (2018)* | EAT-40 | Mean ± SD | 18.8±8.3 | | 15.3±7.4 |
| *Coimbra & Ferreira (2021)* | EDE-Q | Mean ± SD | 1.3±1.2 | 0.9±1.2 | 1.1±1.2 |
| *Collins & Quinton (2020)* | EAT-26 | B (SE) | -0.01 (0.02) | -0.03 (0.01) | Reference group |
| *Dorard & Mathieu (2021)* | EAT-26 | Mean ± SD | 10.5±6.3 | | 12.1±6.9 |
|  |  | N events (%), cutoff ≥20 | 3 (6.1) | | 7 (13.5) |
| *Forestell et al. (2012)* | EAT-26 | Mean ± SD | 16.7±1.5 | | 21.1±2.5 |
| *Gorgoni et al. (2026)* | EDE-Q | Mean ± SD | 1.66±1.41 | | 1.89±1.43 |
| *Gwiozdzik et al. (2022)* | SCOFF | N events (%), cutoff ≥2 | 64 (55.7) | - | 126 (55.5) |
| *Hanras et al. (2022)* | EAT-26 | Mean ± SD | 10.3±7.9 | 8.5±8.0 | 12.2±9.1 |
|  |  | N events (%), cutoff ≥20 | 16 (14.3) | 10 (7.9) | 38 (17.9) |
| *Heiss et al. (2017) ^a^* | EDE-Q | Mean ± SD | - | 1.8±1.2 | 2.2±1.4 |
| *Heiss et al. (2019) ^a^* |  | Mean ± SD | 2.1±1.3 | - | 2.2±1.4 |
| *Köse & Tayfur (2021)* | EAT-40 | N events (%), cutoff ≥31 | 28 (39.4) | | 64 (26.4) |
| *Lindeman, Stark & Latvala (2000) ^b^* | EAT-26 | Mean ± SD | 11.53±13.17 | | 6.01±6.28 |
| *Lindeman M. (2002) ^b^* | EAT-26 | Mean ± SD | 13.86±8.98 | | 9.60±7.32 |
| *McLean et al. (2021)* | EAT-26 | Mean ± SD | - | 10.2±9.1 | 6.8±7.4 |
|  | EDE-Q | Mean ± SD | - | 1.6±1.1 | 1.4±1.1 |
| *McLean et al. (2022) ^c^* | EDE-Q | Mean ± SD | 1.9±1.4 | 1.9±1.5 | 1.7±1.3 |
|  | Self-report diagnosis | n (%) | 33 (11.9) | 78 (13.5) | 35 (4.8) |
| *McLean et al. (2023) ^c^* | EAT-26 | Mean ± SD | 9.8±10.7 | 10.5±11.3 | 8.1±8.9 |
| *Norwood et al. (2019) ^d^* | EDI-3 | Mean (SD) | 1.0 (1.0) | 0.9 (0.9) | 1.1 (1.1) |
| *Paslakis et al. (2020)* | EDE-Q8 | Mean ± SD | 1.3±1.4 | | 1.0±1.3 |
| *Perry et al. (2001)* | Self-report diagnosis | n (%) | 4 (4.3) | | 148 (3.4) |
| *Sieke et al. (2021)* | EDE-Q | Mean ± SD | 1.7±1.4 | | 1.3±1.2 |
|  |  | N events (%), >85th percentile | 22 (16) | | 154 (11) |
| *Timko et al. (2012)* | EAT-26 | Mean ± SD | 10.1±11.6 | 9.2±13.4 | 8.0±8.9 |
| *Zickgraf et al. (2020)* | S-EDE-Q | Mean ± SE | 2.3±0.06 | | 2.1±0.02 |

Notes: All studies are cross-sectional. ^a^ Vegan participants in Heiss et al. (2019) overlapped with those included in Heiss et al. (2017); therefore, only vegetarian participants from the 2019 study were retained to prevent double counting. ^b^ To avoid double counting, only the adolescent sample from Lindeman, Stark, and Latvala (2000) was included, as the adult participants were subsequently reported in Lindeman (2002). ^c^ These studies analyzed different scales measuring eating disorder symptoms within the same study sample. ^c^ Standard deviation was not reported and was assumed to be equal to the mean. Abbreviations: CI, confidence interval; EAT, Eating Attitudes Test; ED, eating disorder; EDDS, Eating Disorder Diagnostic Scale; EDE-Q, Eating Disorder Examination-Questionnaire; EDI, Eating Disorder Inventory; SCOFF, Sick-Control-Fat-Food questionnaire; S-EDE-Q, Short-Eating Disorder Examination-Questionnaire.

**Supplementary Table 4.** Methodological quality of included studies.

| **Reference** | **1** | **2** | **3** | **4** | **5** | **6** | **7** | **8** | **9** | **10** | **11** | **12** | **13** | **14** | **Score** |
| --- | --- | --- | --- | --- | --- | --- | --- | --- | --- | --- | --- | --- | --- | --- | --- |
| Barnett et al. (2016) | Y | Y | CD | Y | N | N | N | NA | Y | N | Y | N | NA | N | 5 |
| Białek-Dratwa et al. (2024) | Y | Y | CD | Y | N | N | N | NA | Y | N | Y | N | NA | N | 5 |
| Çiçekoğlu & Tunçay (2018) | Y | Y | CD | N | N | N | N | NA | N | N | Y | N | NA | N | 4 |
| Coimbra & Ferreira (2021) | Y | Y | CD | Y | N | N | N | NA | N | N | Y | N | NA | N | 4 |
| Collins & Quinton (2020) | Y | Y | CD | Y | N | N | N | NA | Y | N | Y | N | NA | N | 5 |
| Dorard & Mathieu (2021) | Y | Y | CD | N | N | N | N | NA | N | N | Y | N | NA | Y | 4 |
| Forestell et al. (2012) | Y | Y | CD | Y | N | N | N | NA | Y | N | Y | N | NA | N | 5 |
| Gorgoni et al. (2026) | Y | Y | CD | N | N | N | N | NA | Y | N | Y | N | NA | Y | 5 |
| Gwiozdzik et al. (2022) | Y | Y | CD | Y | Y | N | N | NA | N | N | Y | N | NA | N | 5 |
| Hanras et al. (2022) | Y | Y | CD | Y | N | N | N | NA | Y | N | Y | N | NA | N | 5 |
| Heiss et al. (2017) | Y | Y | CD | Y | Y | N | N | NA | Y | N | Y | N | NA | Y | 7 |
| Heiss et al. (2019) | Y | Y | CD | Y | Y | N | N | NA | Y | N | Y | N | NA | Y | 7 |
| Köse & Tayfur (2021) | Y | Y | CD | Y | N | N | N | NA | N | N | Y | N | NA | N | 4 |
| Lindeman, Stark & Latvala (2000) | Y | Y | N | Y | N | N | N | NA | N | N | Y | N | NA | N | 4 |
| Lindeman M. (2002) | Y | Y | Y | Y | N | N | N | NA | N | N | Y | N | NA | N | 5 |
| McLean et al. (2021) | Y | Y | CD | N | Y | N | N | NA | Y | N | Y | N | NA | N | 5 |
| McLean et al. (2022) | Y | Y | Y | Y | N | N | N | NA | N | N | Y | N | NA | N | 5 |
| McLean et al. (2023) | Y | Y | Y | Y | N | N | N | NA | N | N | Y | N | NA | N | 5 |
| Norwood et al. (2019) | Y | Y | Y | Y | N | N | N | NA | Y | N | Y | N | NA | N | 6 |
| Paslakis et al. (2020) | Y | Y | N | Y | N | N | N | NA | Y | N | Y | N | NA | N | 5 |
| Perry et al. (2001) | Y | Y | Y | Y | N | N | N | NA | Y | N | Y | N | NA | Y | 7 |
| Sieke et al. (2021) | Y | Y | CD | Y | N | N | N | NA | Y | N | Y | N | NA | Y | 6 |
| Timko et al. (2012) | Y | Y | Y | Y | N | N | N | NA | N | N | Y | N | NA | N | 5 |
| Zickgraf et al. (2020) | Y | Y | N | Y | N | N | N | NA | N | N | Y | N | NA | Y | 5 |

Notes: Each cross-sectional study was rated as good (most criteria met), fair (some criteria met), or poor (few criteria met) according to the National Institutes of Health’s Quality Assessment Tool for Observational Cohort and Cross-Sectional quality rating guide. Numbers represent the questions included in the National Institutes of Health’s Quality Assessment Tool for Observational Cohort and Cross-Sectional Studies: 1. Question clear? 2. Population clearly defined? 3. >50% participants? 4. Recruitment populations consistent? 5. Sample size justified? 6. Exposure assessed prior to outcome? 7. Sufficient timeframe? 8. Different exposure levels? 9. Valid exposure? 10. Repeated exposure assessment? 11. Valid outcomes? 12. Outcome assessors blinded? 13. Loss to follow up <20%? 14. Confounders adjusted for? Item 8 was considered not applicable for dichotomous exposure. Item 13 was considered not applicable for cross sectional studies. Abbreviations: CD: cannot determine; N, no; NA, not applicable; NR, not reported; Y, yes.

**Supplementary Table 5.** Subgroup analyses for the cross-sectional associations between vegetarian and vegan vs. omnivorous diets and eating disorder symptoms.

| **Subgroups** | **n** | **SMD (95% CI)** | **p** | **I^2^ (%)** | **p _subgroup_** |
| --- | --- | --- | --- | --- | --- |
| **Adolescents and adults** |  |  |  |  |  |
| **Diet type ^a^** |  |  |  |  | 0.524 |
| Vegetarian (vs. omnivorous) | 9 | 0.024 (-0.088, 0.137) | 0.632 | 40.3 |  |
| Vegan (vs. omnivorous) | 9 | -0.050 (-0.291, 0.196) | 0.650 | 86.7 |  |
| **Measurement scale ^b^** |  |  |  |  | 0.392 |
| EAT | 12 | 0.154 (-0.057, 0.366) | 0.135 | 83.2 |  |
| EDE-Q ^c^ | 10 | 0.055 (-0.094, 0.204) | 0.427 | 80.1 |  |
| **Region ^d^** |  |  |  |  | 0.633 |
| Europe | 8 | 0.106 (-0.168, 0.379) | 0.391 | 82.1 |  |
| North America | 8 | 0.044 (-0.156, 0.245) | 0.620 | 80.5 |  |
| Oceania | 4 | -0.051 (-0.556, 0.455) | 0.709 | 89.5 |  |
| **Adults** | **n** | **SMD (95% CI)** | **p** | **I^2^ (%)** | **p _subgroup_** |
| **Diet type ^a^** |  |  |  |  | 0.660 |
| Vegetarian (vs. omnivorous) | 8 | 0.037 (-0.089, 0.163) | 0.509 | 41.1 |  |
| Vegan (vs. omnivorous) | 8 | -0.019 (-0.296, 0.257) | 0.872 | 94.5 |  |
| **Measurement scale ^b^** |  |  |  |  | 0.628 |
| EAT | 9 | 0.111 (-0.108, 0.330) | 0.276 | 80.1 |  |
| EDE-Q ^c^ | 10 | 0.055 (-0.094, 0.204) | 0.427 | 80.1 |  |
| **Region ^d^** |  |  |  |  | 0.848 |
| Europe | 6 | -0.031 (-0.232, 0.171) | 0.709 | 75.0 |  |
| North America | 7 | 0.039 (-0.192, 0.270) | 0.694 | 83.2 |  |
| Oceania | 2 | 0.011 (-2.105, 2.128) | 0.957 | 86.0 |  |

Notes: Results are presented for studies including both adolescent and adult populations. ^a^ Studies that did not report data on vegetarians or vegans separately were excluded from the analysis. ^b^ Scales represented by ≤2 studies (i.e., EDI and SCOFF) were excluded from the analysis. ^c^ Studies included only adult populations. ^d^ Europe: France, Germany, Poland, and Portugal; North America: USA; and Oceania: Australia. Studies that included populations from multiple countries without reporting region-specific results were excluded from the analysis. Regions represented by ≤2 studies (i.e., Asia [Turkey]) were also excluded. Abbreviations: CI, confidence interval; EAT, Eating Attitudes Test; EDE-Q, Eating Disorder Examination-Questionnaire; SMD, standardized mean difference.

**Supplementary Table 6.** Meta-regression analyses for the cross-sectional associations between vegetarian and vegan vs. omnivorous diets and eating disorder symptoms.

| **Covariates** |  | **Coefficients** | | | **Heterogeneity** | | | **Residual heterogeneity** | |
| --- | --- | --- | --- | --- | --- | --- | --- | --- | --- |
| **Adolescents and adults** | **n** | **β** | **SE** | ***p*** | ***I*^2^ (%)** | **H^2^** | **R^2^ (%)** | **Q** | ***p*** |
| Age (mean, years) | 21 | -0.006 | 0.008 | 0.478 | 88.3 | 8.5 | 0 | 103.7 | <0.001 |
| Sex (% female) | 23 | -0.004 | 0.004 | 0.311 | 87.1 | 7.8 | 0 | 96.1 | <0.001 |
| Total sample size (n) | 23 | 0.000 | 0.000 | 0.726 | 84.9 | 6.6 | 0 | 107.5 | <0.001 |
| BMI (mean, kg/m^2^) | 16 | 0.033 | 0.054 | 0.551 | 85.3 | 6.8 | 0 | 86.3 | <0.001 |
| Underweight status (%) | 10 | 0.041 | 0.022 | 0.105 | 75.6 | 4.1 | 24.5 | 20.8 | 0.004 |
| Publication year | 23 | -0.017 | 0.008 | 0.057 | 85.7 | 7.01 | 13.44 | 108.2 | <0.001 |
| **Adults** | **n** | **β** | **SE** | ***p*** | ***I*^2^ (%)** | **H^2^** | **R^2^ (%)** | **Q** | ***p*** |
| Age (mean, years) | 17 | -0.002 | 0.008 | 0.855 | 86.2 | 7.3 | 0 | 81.9 | <0.001 |
| Sex (% female) | 19 | -0.008 | 0.003 | 0.028 | 81.2 | 5.3 | 20.6 | 72.1 | <0.001 |
| Total sample size (n) | 19 | 0.000 | 0.000 | 0.499 | 81.6 | 5.4 | 0 | 84.7 | <0.001 |
| BMI (mean, kg/m^2^) | 15 | 0.045 | 0.055 | 0.432 | 85.1 | 6.7 | 0 | 76.0 | <0.001 |
| Underweight status (%) | 9 | 0.062 | 0.031 | 0.095 | 74.9 | 4.0 | 28.9 | 14.6 | 0.023 |
| High educational level (%) | 13 | 0.002 | 0.002 | 0.483 | 84.5 | 6.5 | 0 | 48.1 | <0.001 |
| Publication year | 19 | 0.002 | 0.016 | 0.929 | 85.3 | 6.8 | 0 | 88.5 | <0.001 |

Notes: Results are presented separately for studies including adolescent and adult populations and for studies restricted to adult populations. Univariate random-effects meta-regression models were used to estimate the proportion of between-study heterogeneity explained by each covariate, as well as the change in the effect size estimate for each 1-unit change in the characteristic included as predictor in the model. n represents the number of studies included in each meta-regression model. Abbreviations: BMI, body mass index; H^2^, unaccounted variability; I^2^, residual heterogeneity; Q, Cochran’s Q statistic; SE, standard error; R^2^, proportion of heterogeneity explained.

**Supplementary Table 7.** Sensitivity analyses excluding studies one by one.

| **Reference removed** | **SMD** | **LL** | **UL** | **I^2^ (%)** |
| --- | --- | --- | --- | --- |
| All studies | 0.074 | -0.044 | 0.192 | 80.3 |
| Barnett et al. (2016) | 0.0709 | -0.0506 | 0.1925 | 81.2 |
| Białek-Dratwa et al. (2024) | 0.0725 | -0.0513 | 0.1963 | 81.2 |
| Çiçekoğlu & Tunçay (2018) | 0.0632 | -0.0559 | 0.1823 | 80.9 |
| Coimbra & Ferreira (2021) | 0.0806 | -0.0424 | 0.2036 | 81.1 |
| Collins & Quinton (2020) | 0.0885 | -0.0324 | 0.2094 | 79.3 |
| Dorard & Mathieu (2021) | 0.0854 | -0.0344 | 0.2053 | 80.8 |
| Forestell et al. (2012) | 0.086 | -0.0343 | 0.2062 | 80.7 |
| Gwiozdzik et al. (2022) | 0.0874 | -0.0342 | 0.2089 | 79.5 |
| Gorgoni et al. (2026) | 0.0783 | -0.0456 | 0.2022 | 81.1 |
| Hanras et al. (2022) | 0.0924 | -0.0259 | 0.2107 | 77.5 |
| Heiss et al. (2017) | 0.0948 | -0.0212 | 0.2108 | 76.5 |
| Heiss et al. (2019) | 0.0796 | -0.0435 | 0.2027 | 81.1 |
| Köse & Tayfur, (2021) | 0.0588 | -0.0594 | 0.1771 | 80.5 |
| Lindeman, Stark & Latvala (2000) | 0.0552 | -0.0565 | 0.1669 | 80.2 |
| Lindeman M. (2002) | 0.0528 | -0.0614 | 0.1669 | 79.8 |
| McLean et al. (2021) | 0.0629 | -0.0591 | 0.1849 | 80.5 |
| McLean et al. (2022/2023) | 0.0698 | -0.0547 | 0.1942 | 80.0 |
| Norwood et al. (2019) | 0.0867 | -0.0344 | 0.2078 | 80.4 |
| Paslakis et al. (2020) | 0.0661 | -0.0571 | 0.1893 | 80.7 |
| Perry et al. (2001) | 0.074 | -0.0487 | 0.1967 | 81.2 |
| Sieke et al. (2021) | 0.0602 | -0.0609 | 0.1813 | 79.8 |
| Timko et al. (2012) | 0.0686 | -0.0552 | 0.1923 | 81.0 |
| Zickgraf et al. (2020) | 0.0717 | -0.053 | 0.1964 | 80.8 |

Notes: Results are presented for studies including both adolescent and adult populations. Abbreviations: LL, low limit; SMD, standardized mean differences; UL, upper limit.

**Supplementary Figure 1.** Pooled standardized mean differences in eating disorder symptoms comparing vegetarian and vegan with omnivorous diets in studies including adult populations.

Abbreviations: CI, confidence interval; EAT, Eating Attitudes Test; EDE-Q, Eating Disorder Examination-Questionnaire; EDI, Eating Disorder Inventory; HK, Hartung-Knapp; OMN, omnivorous; SCOFF, Sick-Control-Fat-Food questionnaire; S-EDE-Q, Short-Eating Disorder Examination-Questionnaire; SMD, standardized mean difference; VEG, vegetarians and vegans.

**D**

**A**

**B**

**C**

**F**

**E**

**Supplementary Figure 2.** Meta-regression models by age (A), females (B), total sample size (C), body mass index (D), underweight status (E), and publication year (F) for studies including both adolescent and adult populations.

Notes: A) Mean age (years); B) Percentage of females; C) total sample size; D) Body mass index (mean, kg/m^2^); and E) Percentage of underweight status (BMI <18 kg/m^2^).

**A**

**B**

**D**

**C**

**F**

**E**

**G**

**Supplementary Figure 3.** Meta-regression models by age (A), females (B), total sample size (C), body mass index (D), underweight status (E), educational level (F) and publication year (G) for studies restricted to adult populations. Notes: A) Mean age (years); B) Percentage of females; C) total sample size; D) Body mass index (mean, kg/m2); E) Percentage of underweight status (BMI <18 kg/m2); (F) Percentage of high educational level (university degree); and (G) Publication year.

**Supplementary Figure 4.** Funnel plot of publication bias.

Notes: Egger’s test for funnel plot asymmetry: t = –0.31, df = 21, *p* = 0.758. Results are presented for studies including both adolescent and adult populations.
